# How to Preserve Pills of the Extract of Aloes

**Published:** 1845-10

**Authors:** 


*How to Preserve Pills of Extract of Aloes.*—Extract of aloes readily absorbs moisture from the atmosphere, which renders it difficult to preserve in the form of pills. This inconvenience may be perfectly avoided, according to M. Rottscher, by adding a fourth part of the carbonate of magnesia. *Lond. and Edin. Monthly Jour. of Med. Sci.—Ibid.*

